# Light Stable Isotope Ratios of US Pureed Baby Foods

**DOI:** 10.1002/rcm.10119

**Published:** 2025-08-09

**Authors:** Kirsten A. Verostick, Alli Randall, Chris Stantis, Stephannie Covarrubias, Gabriel J. Bowen

**Affiliations:** ^1^ Department of Geology and Geophysics University of Utah Salt Lake City Utah USA; ^2^ School of Anthropology, Political Science, and Sociology Southern Illinois University Carbondale Illinois USA

**Keywords:** biogeochemical methods, infant feeding practices or infant diet, stable isotopes

## Abstract

**Rationale:**

The isotopic composition of foods is useful for verifying origin and provides baseline information for interpreting isotopic data from human tissues in dietary and forensic research. Despite their widespread consumption, baby foods in the United States remain isotopically understudied. This study presents and analyzes an exploratory δ^2^H, δ^18^O, δ^13^C, and δ^15^N dataset for pureed baby foods spanning different food types, labeling designations, brands, and geographic purchase locations.

**Methods:**

Pureed baby food was collected from across the United States, focusing on three widely consumed food types (banana, carrot, and sweet potato, *n* = 117) across several commercial brands. Cryogenically extracted water and residual dry solids were analyzed to determine the δ^2^H and δ^18^O values of water and δ^13^C and δ^15^N values of bulk solids.

**Results:**

We found significant isotopic differences between food types, with bananas having the highest δ^2^H, δ^18^O, and δ^13^C values and carrots the lowest. These offsets persisted across different brands. No significant differences were observed across purchase locations. δ^15^N values were significantly higher for bananas and carrots labeled “organic,” and d‐excess and δ^18^O values in banana and sweet potatoes were significantly higher for foods containing added water.

**Conclusions:**

Our dataset documents isotopic patterns in pureed baby foods aligning with expectations related to differences in growing environment, farming practices (e.g., organic), and processing (e.g., added water). This supports the potential utility of stable isotope data for studying and authenticating baby food production and distribution. Although limited in sample size, the absence of geographic isotopic variation, which is consistent with geographic homogenization of the modern “supermarket diet,” suggests isotopic inputs from food to bodies of infants and children consuming these prepared foods depend more on consumer choices than on location. Thus, early childhood isotope data may be more informative for reconstructing diet than residence.

## 1 Introduction

Understanding early childhood dietary patterns is essential for both nutritional research and forensic investigations, particularly in cases of malnutrition, neglect, or geographic origin tracing. In the United States, commercial and homemade pureed baby foods play a significant role in infant diets. Data from the Feeding Infants and Toddlers Studies (FITS) indicate that among US children aged 6 months to 1 year, pureed baby food accounts for approximately 52% of vegetable intake and 44% of fruit intake, with carrots and sweet potatoes being the most commonly consumed vegetables [1, 2]. Given their widespread consumption, analyzing these foods provides valuable insight into early dietary exposures, nutritional quality, and potential forensic applications.

Isotope analyses are foundational across scientific fields, used in ecology, archaeology, forensic science, and environmental studies to offer insights on biological and environmental processes. Isotopic systems, like oxygen (δ^18^O), nitrogen (δ^15^N), hydrogen (δ^2^H), carbon (δ^13^C), sulfur (δ^34^S), strontium (^87^Sr/^86^Sr), and lead (^206^Pb/^207^Pb), offer information about human diet and geographical origin [3, 4, 5, 6, 7, 8, 9, 10]. Forensic analyses frequently rely on isotopic signatures from human tissues, such as hair, fingernail, bone (apatite and collagen), and teeth samples, as each provides a different temporal perspective relevant to casework. For instance, the first molar's crown, which is often sampled for both stable and radiogenic isotopes in forensic casework, forms approximately between the ages of 2 months to 3 years old [11], embedding the isotopic signatures of water and foods consumed during this developmental period, which can be informative in both forensic and dietary interpretations. Establishing a baseline understanding of isotopic variation in baby foods can aid in dietary and forensic investigations, improving our ability to interpret isotopic patterns in human tissues.

Despite the potential utility of such data for dietary studies, forensic applications [3], and food origin verification [12, 13], no comprehensive isotopic reference dataset exists for baby foods in the United States. Although some studies have analyzed baby foods using mass spectrometry, most have focused on nutrient content (e.g., essential elements and fatty acids) or the presence of harmful substances (e.g., acrylamide, mycotoxins, toxic metals, and volatile organic compounds) [14, 15, 16, 17, 18, 19, 20]. Few have examined isotopic compositions of specific foods, and those that have provide only generalized findings with limited applicability to forensic and dietary research [14]. This gap underscores the need for systematic isotopic characterization of commonly consumed baby foods.

Our research sought to (1) characterize isotopic differences across baby food types, (2) assess regional variations in isotopic values across US sources, (3) explore differences across brands, and (4) determine if labeling designations, such as “added water” or “organic,” are associated with measurable isotopic differences. These objectives should establish a baseline for understanding and applying isotopic variation in pureed baby foods in dietary and forensic research.

## 2 Materials and Methods

### 2.1 Materials

A total of 133 food samples were purchased from grocery stores across the United States. We use the term “flavor” to refer to the type of pureed food, as determined by the primary ingredient (e.g., specific fruit, vegetable, or a mix thereof) it contains. Our sampling focused on the representation of three widely consumed flavors (banana, carrots, and sweet potatoes) across several brands and with a variety of labeling designations (organic, natural, Stage 1, etc.).

### 2.2 Methods: Stable Isotope Analysis

Sample preparation and analysis were conducted at the Stable Isotope Ratio Facility for Environmental Research (SIRFER) at the University of Utah (Salt Lake City, United States). Isotopic values are reported in delta notation (*δ* = [*R*_*sample*_ − *R*_*standard*_] / *R*_*standard*_, where *R* is the ratio of rare to common isotope abundance) and units of per mil (‰, parts per thousand) [21, 22, 23].

Water was extracted from baby food samples via vacuum distillation [24] to perform δ^2^H and δ^18^O analysis. After extraction, activated charcoal was added for a 48‐h period to adsorb organic compounds. The water samples were then transferred to gas chromatography vials for δ^2^H and δ^18^O analysis using a Picarro L2130‐i Cavity Ring‐Down Spectrometer (Santa Clara, CA [25]). Data were screened using the ChemCorrect software (Picarro Inc.) to identify any spectral interference associated with volatile organic compounds that may have been extracted from the samples; none was observed. Measurements of three laboratory reference waters were included to correct raw data for sample‐to‐sample memory effects and run drift (PZ: 18.1‰, 1.93‰; EV: −72.3‰, −10.16‰; and UT2: −119.1‰, −15.84‰; for δ^2^H and δ^18^O, respectively). Data were averaged to obtain uncalibrated sample values, and values for the UT2 and PZ waters were used to calibrate sample values against VSMOW2 (Vienna Standard Mean Ocean Water 2) and GISP (Greenland Ice Sheet Precipitation) using a two‐point linear calibration. Analytical precision for the Picarro analysis is approximately 0.31‰ for δ^2^H and 0.05‰ for δ^18^O.

Following water extraction, the residual solids were analyzed for δ^13^C and δ^15^N values. Approximately 1 mg of sample was weighed into 3.5 × 5 mm tin capsules. Approximately 10% of samples were run in duplicate, and values for these samples were averaged and reported as a single value in the supplementary data table. δ^13^C and δ^15^N analysis was conducted using a Thermo Finnigan Delta Advantage continuous‐flow isotope ratio mass spectrometer coupled to a Carlo Erba CHN EA1110 elemental analyzer via a Thermo Finnigan Conflo III device [26]. Internal laboratory reference materials with known isotopic values (UU‐CN‐1 [glutamic acid]: 49.61‰, 23.96‰; UU‐CN‐2 [glutamic acid]: −4.56‰, −28.18‰; and Spinach: −0.4‰, −27.41‰; for δ^15^N and δ^13^C, respectively) were analyzed alongside the samples and used to correct for instrumental drift. Values for UU‐CN‐1 and UU‐CN‐2 were used to calibrate the data to the VPDB (δ^13^C) and AIR (δ^15^N) scales, as defined by the USGS40 and USGS41 international standards, using a 2‐point linear calibration. Analytical precision was within ± 0.2‰ standard deviation for both isotopes, based on repeated measurement of internal laboratory standards and secondary reference materials.

### 2.3 Methods: Statistical Analysis

All statistical analyses were conducted using R Software Version 4.3.1. We used ANOVA to test for isotope effects for categorical variables with > 2 levels and *t* tests to test for differences in means where only two groups were compared and for post hoc comparisons.

## 3 Results and Discussion

The complete table of results from the 133 samples is available in the Supporting Information. A total of 117 samples from our three focus flavors (bananas, carrots, and sweet potatoes) were collected, representing nine different commercial brands (Table 1). The remaining 16 samples were assorted blends of fruits and vegetables and are reported but not further discussed.

**TABLE 1:** Distribution of brands among flavors.

Flavor→			
Brand ↓	Banana	Carrot	Sweet potato
Beech‐Nut	14	11	11
Earth's Best Organic	1	3	3
Gerber	20	15	15
Happy Baby	0	4	4
Meijer	1	1	1
Parent's Choice	4	3	3
Plum Organics	0	0	1
Simple Truth Organic	1	0	0
Tippy Toes	1	0	0
Total count	42	37	38

### 3.1 Flavor

**FIGURE 1:**
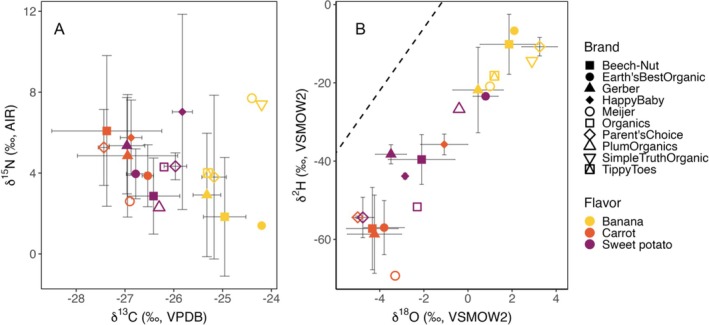
(A) Mean +1SD carbon (δ^13^C) and nitrogen (δ^15^N) isotopic values for flavors by brand. (B) Mean +1SD oxygen (δ^18^O) and hydrogen (δ^2^H) isotopic values for flavors by brand; dashed line is global meteoric water line (GMWL, δ^2^H = 8 × δ^18^O), which describes the relationship between H and O δ‐values in global precipitation and approximates the expected values for unevaporated meteoric waters.

Statistical analysis reveals significant isotopic differences among flavors for all four of the measured isotopic systems (carbon: *F*[2, 112] = 93.47, *p* < 0.001; oxygen: *F*[2, 112] = 113.4, *p* < 0.001; hydrogen: *F*[2, 112] = 145.9, *p* < 0.001; and nitrogen: *F*[2, 112] = 6.52, *p* = 0.002). Isotope *δ* values for H, O, and C vary systematically among the three food flavors (Figure 1) and clearly differentiate the banana flavor group, which shows little to no overlap with either the carrot or sweet potato samples. In our samples, bananas exhibited significantly higher δ^2^H values than carrots (*t* = 16.07, df = 73.37, *p* < 0.001) and sweet potatoes (*t* = 10.30, df = 75.93, *p* < 0.001), higher δ^18^O values than carrots (*t* = 15.78, df = 74.79, *p* < 0.001) and sweet potatoes (*t* = 9.79, df = 68.82, *p* < 0.001), and higher δ^13^C values than carrots (*t* = 11.89, df = 50.06, *p* < 0.001) and sweet potatoes (*t* = 12.54, df = 68.99, *p* < 0.001). Sweet potatoes and carrots exhibit stronger overlap, but average values for carrots are significantly lower than those for sweet potatoes for all three isotope ratios (δ^2^H: *t* = −7.12, df = 68.74, *p* < 0.001; δ^18^O: *t* = −4.10, df = 69.24, *p* < 0.001; and δ^13^C: *t* = −3.24, df = 58.22, *p* = 0.002). There is strong δ^15^N overlap between flavors, but a slightly lower mean value is observed for bananas than either carrots (*t* = −3.42, df = 75.87, *p* = 0.001) or sweet potatoes (*t* = −2.19, df = 75.44, *p* = 0.03).

All three flavors are derived from plants that use the C_3_ photosynthetic pathway, which discriminates strongly against ^13^C during photosynthesis [27, 28, 29, 30]. The values observed align with the expected range for C_3_ plants, from −22‰ to −30‰ [31, 32, 33, 34], consistent with the foods' labeling indications that report few or no added ingredients (e.g., sugars and stabilizers) that might be derived from C_4_ plants (Figure 1A). Environmental conditions can exert a strong influence on the magnitude of photosynthetic carbon isotope fractionation in C_3_ plants, and in particular, plants subjected to higher levels of water stress tend to have higher δ^13^C values [35, 36, 37]. Bananas, which are grown in tropical and subtropical climates and are commonly subjected to drought stress [35], stand out among our samples for their relatively high δ^13^C values. These values are within the range previously reported for unprocessed banana fruits [38]. Carbon isotopic differences between carrots and sweet potatoes are more modest but also align with growing conditions, with sweet potatoes, which are commonly grown in warmer climates, having higher values than carrots.

Nitrogen isotopic values in plants can be strongly influenced by fertilizers [33], which are further discussed in Section 3.4, and by natural differences in nitrogen cycling between ecosystems. In particular, greater plant uptake of NO_3_^−^, which is typically ^15^N‐depleted relative to other soil nitrogen sources, has been proposed in tropical and subtropical water environments [39] and could explain the slightly lower δ^15^N values measured here for bananas (Figure 1A).

The water in undiluted fruit and vegetable products originates from the plants and reflects the isotopic values of plant water during growth. The higher δ^2^H and δ^18^O values observed in banana‐flavored baby food (Figure 1B) are consistent with the higher values that characterize rainfall (and thus soil, ground, and irrigation water) in tropical and subtropical regions [23, 29]. Similarly, the higher mean values for sweet potatoes are consistent with the production of this crop in somewhat warmer environments than carrots. Thus, the first‐order patterns are consistent with a geographic control on food water δ^2^H and δ^18^O values resulting from differences in the isotope ratios of growth waters.

All samples also show isotopic evidence for evaporation; however, in the form of deuterium excess values (*d* = δ^2^H—8 x δ^18^O) well below the typical value for continental fresh waters (+10‰), which can vary subtly between locations due to hydroclimatic processes but seldom approach the low values observed here [40, 41]. This evaporation could occur at a number of points in the growth and production of the foods, including evaporation of soil or irrigation waters before uptake by crops or evaporation from the fruits and vegetables during growth or following harvest. Although it is not possible to distinguish between these alternatives based on the isotopic data alone, we note that evaporative effects are strongest in the bananas (which have the lowest *d* values), suggesting enhanced evaporative losses in these warm‐climate fruits. This is broadly consistent with our interpretation (above) of a water stress influence on the δ^13^C values of bananas. Regardless of the mechanisms, both the water isotopes (δ^2^H and δ^18^O) and δ^13^C values suggest that isotopic expressions of growth environments and conditions are preserved in the baby foods and are not compromised during preparation and processing of the foods.

### 3.2 Added Water

**FIGURE 2:**
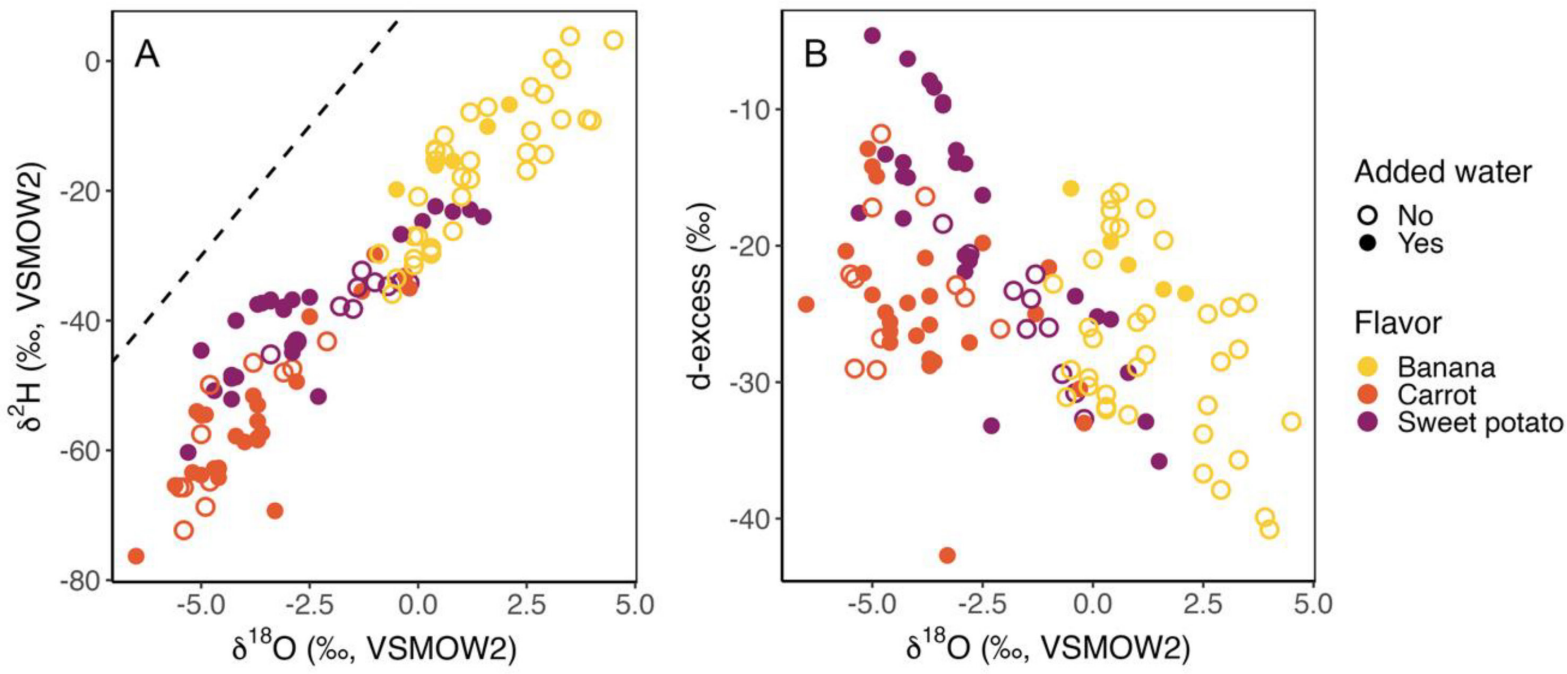
(A) Biplot of δ^18^O and δ^2^H values of individual samples, grouped by flavor and presence of added water. The dashed line represents the GMWL. (B) Biplot of δ^18^O and d‐excess values for individual samples, grouped by flavor and presence of added water.

Although many samples consist solely of pureed fruit or vegetable, several list added water in their ingredients. H and O isotope data have previously been used to detect the presence of added water in foods and beverages [13, 42, 43, 44, 45, 46]. This is possible because, unlike the natural water contained in fruits and vegetables, added water typically has high *d* values (close to +10‰) and shifts the isotopic composition of the bulk water in the food products toward higher values of *d*. Our collection includes samples both with and without added water for all three flavors, although the prevalence of water addition varies substantially for different flavors and brands. Significant differences in *d*‐excess values between samples with and without added water were noted in sweet potatoes (*t*[29.61] = −3.37, *p* = 0.002) and bananas (*t*[10.11] = −3.86, *p* = 0.003), although the number of bananas with added water samples was small (*n* = 5). In both cases, samples labeled as containing added water exhibited higher *d*‐excess values, averaging −18.0‰ (SD = 8.43) for sweet potatoes and −20.7‰ (SD = 3.15) for bananas, consistent with the expectation of dilution (Figure 2). We suggest that these results indicate added water can measurably affect the isotopic composition of water in baby foods. Consequently, isotope ratios of water may be useful to detect the use of this processing step, but the water isotope values transferred to babies who consume these foods will be complicated by the blend of plant‐derived and added waters that they contain.

### 3.3 Locations and Brands

Our samples included baby foods purchased at ~22 different locations across the contiguous United States. No significant isotopic differences between purchase locations were found across the entire dataset or within individual brands or flavors. The absence of regional variation is consistent with the “supermarket diet” concept [47, 48], which posits that regional variation in dietary isotope ratios is strongly damped or eliminated by the wide array of nonlocal foods and beverages readily available to consumers today.

In contrast, we observed a significant effect of brand on the δ^13^C values of sweet potatoes (*F*[6, 30] = 5.566, *p* < 0.001) and bananas (*F*[6, 34] = 5.156, *p* < 0.001), the δ^18^O values of carrots (*F*[5, 31] = 6.298, *p* < 0.001), sweet potatoes (*F*[6, 30] = 19.49, *p* < 0.001), and bananas (*F*[6, 34] = 4.282, *p* < 0.001), and the δ^2^H values for carrots (*F*[5, 20] = 6.99, *p* < 0.001), sweet potatoes (*F*[6, 28] = 48.25, *p* < 0.001), and bananas (*F*[6, 31] = 4.414, *p* = 0.01). No significant brand influence on δ^15^N values was observed for any of the flavors. Carbon isotope *δ* values for specific brands of a given flavor typically fell within a narrow range, with most brand‐flavor combinations having a standard deviation ≤ 0.5‰ (Figure 1). Two widely available brands of carrots (Gerber and Beech‐Nut) are anomalous in this respect, with 1*σ* values exceeding 1‰. The dispersion of δ^2^H and δ^18^O values for individual brand‐flavor combinations is somewhat more variable but is generally small relative to the range of values spanning brands and flavors.

Collectively, this evidence suggests that differences in the locations and/or conditions of production for the fruits and vegetables used by different brands may be reflected in the isotope values of the foods. In some cases, these may be interpretable based on brand information. For example, the Happy Baby brand is a product of Poland, and their carrots have higher δ^18^O values than any of the US sourced brands, which may reflect differences in the isotopic composition of the rainfall and/or irrigation water at the different sites of growth. In general, however, most brands provide limited public information on the source of their ingredients or provide descriptions implying that their sources vary over time. In this respect, it is interesting that we did observe substantial brand‐specific isotopic differentiation, implying that even if sources for individual brands are dynamic, the variation within given brands was smaller than that between brands (at least over the scales sampled in our study). This highlights the potential for isotopes to provide information on baby food ingredient source and the supply chain in forensic and food science studies.

### 3.4 Labeling Designations

**FIGURE 3:**
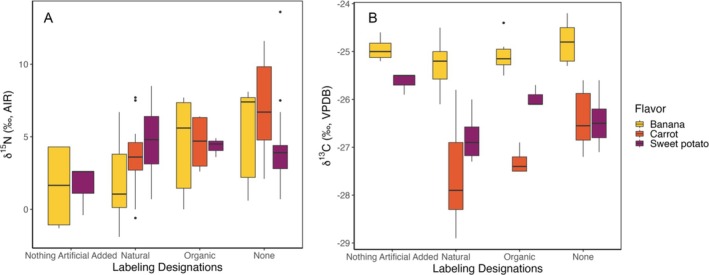
(A) Boxplot of δ^15^N values for each flavor by labeling designations. (B) Boxplot of δ^13^C values for each flavor by labeling designations.

The different pureed foods presented a number of different designations on their labels, including “organic,” “natural,” and age stage designations (e.g., Stage 1: 4 months+, supported sitter first foods). We compared the δ^13^C and δ^15^N values across different labeling categories that may relate to farming and production practices, including “organic,” “natural,” and “nothing artificial added” (Figure 3). The “organic” designation was found on products labeled as 100% USDA organic, verified by third party companies such as Oregon Tilth or QAI. We would expect “organic” foods to have higher δ^15^N values due to the use of manure, compost, and/or amino acid–based fertilizers based on previous research on nitrogen isotopes in plants and soil [33, 49, 50, 51, 52, 53]. The FDA informally defines “natural” as containing no synthetic substances, such as artificial colors or flavors [54]. “Nothing artificial added” is an informal designation often considered synonymous with “natural.” All flavors, regardless of designation, were labeled as “non‐GMO verified.” The primary additives listed across the foods, regardless of designation, included lemon juice (derived from a C_3_ plant [55]) and ascorbic acid/vitamin C (typically derived from C_3_ plant sources [56]). Thus, we would expect all the samples to exhibit δ^13^C values consistent with those expected for C_3_ plants unless they contained unlisted additives (i.e., C_4_ sugars).

We found significant differences in the δ^15^N values as a function of designation across all flavors (*F*[3, 111] = 7.45, *p* < 0.001) and significant differences within flavors for bananas (*F*[3, 37] = 3.803, *p* = 0.02), and carrots (*F*[2, 34] = 7.011, *p* < 0.001). For both bananas and carrots, the highest average δ^15^N values were associated with foods labeled “organic” (Figure 3A). Although not always observed in previous studies, the use of organic fertilizers has the potential to increase the δ^15^N values of food crops, consistent with our results for bananas and carrots [33]. Organic‐designated sweet potatoes, in contrast, did not exhibit higher δ^15^N values (Figure 3A). Sweet potatoes with the “natural” designation had the lowest average δ^13^C values and highest average δ^15^N values (Figure 3B), but the data showed significant variation within and overlap between designations. Sweet potatoes are sensitive to growing conditions, and water‐stressed or drought conditions have been shown to decrease their δ^15^N and increase δ^13^C values [37]; the variation seen in our data may reflect differences in growing environment rather than systematic effects of different farming practices.

Statistically significant differences in δ^13^C values were found across designations (*F*[3, 111] = 3.095, *p* = 0.03), but the differences between these groups were small relative to the differences between flavors. In general, δ^13^C values for samples with no designation or designated “natural” tended to be lower than those labeled “organic” or “nothing artificial added” (Figure 3B). As discussed above, the “natural” and “nothing artificial added” designations both imply the absence of additives, and the lack of systematic δ^13^C differences between these samples and the others, as well as the differences observed between the “natural” and “nothing artificial added” samples for banana and sweet potato flavors, suggests that food additives did not systematically or measurably affect the δ^13^C values of the samples. Unlike the other designations, “organic” is associated with specific farming and production practices. Some previous studies have reported δ^13^C differences between organically and conventionally grown crops [57, 58, 59], whereas others found no such differences [38, 60] or variable patterns for different crop types [33, 61]. The underlying drivers of these variations remain unclear. Differences could arise processes such as regulation of leaf‐level gas exchange [58] or activity levels of RuBisCo enzyme [57, 59]. Given that our dataset is relatively small, and we are not able to account for other variables like location, growing season, and environmental conditions of crop production that might also influence the δ^13^C values of the fruits and vegetables, we suggest that the observed δ^13^C differences among designations are worth noting but may not be systematic or predictable.

## 4 Conclusions

New stable isotope data (δ^2^H, δ^13^C, δ^15^N, and δ^18^O values) for three common US baby food flavors (bananas, carrots, and sweet potatoes) show distinct isotopic patterns among flavors, brands, added water, and labeling designations. Many of these patterns can be attributed to known mechanisms linking the isotopic composition of food products to plant growth environment, farming practices, and food production processes. These new data provide a reference and context for food science and forensic applications, particularly those involving dietary origin and/or supply chain analyses. The results imply that pureed baby foods, like other components of the modern supermarket diet, are unlikely to transfer locally or regionally characteristic isotope signatures to consumers and that their consumption may therefore complicate attempts to geolocate human remains based on isotopic data. Expanding this dataset to include other commonly consumed pureed foods and infant formulas may further enhance the existing reference framework for the use of isotopes in forensic and dietary research involving young children.

## Author Contributions

**Kirsten A. Verostick:** conceptualization, investigation, writing – original draft, methodology, validation, visualization, writing – review and editing, software, formal analysis, data curation. **Alli Randall:** conceptualization, investigation, writing – original draft, writing – review and editing, methodology, formal analysis. **Chris Stantis:** conceptualization, writing – review and editing, methodology. **Stephannie Covarrubias:** conceptualization, writing – review and editing. **Gabriel J. Bowen:** writing – original draft, writing – review and editing, validation, project administration, funding acquisition, resources, supervision, methodology, conceptualization.

## Peer Review

The peer review history for this article is available at https://www.webofscience.com/api/gateway/wos/peer‐review/10.1002/rcm.10119.

## Supporting information


**Data S1:** Supporting Information.

## Data Availability

The data that support the findings of this study are available in the Supporting Information.
